# Case Report: Application of combined transplantation of multiple autologous flaps in preventing empty pelvis syndrome after radical surgery for isolated sacrococcygeal recurrence of anal canal squamous

**DOI:** 10.3389/fsurg.2025.1705860

**Published:** 2025-12-03

**Authors:** Xiaoling Jiang, Lixia Zhang, Haixia Shu, Min Wang, Shengyan Xie, Xiaojuan Wang, Lianli He

**Affiliations:** Department of Gynecology and Obstetrics, The Third Affiliated Hospital of Zunyi Medical University (The First People's Hospital of Zunyi), Zunyi, Guizhou, China

**Keywords:** anal canal tumor, rectovaginal fistula, hypercalcemia, surgical flap, thin femoris muscle

## Abstract

Anal canal carcinoma is a rare malignancy, accounting for less than 3% of gastrointestinal tumors. Recurrent anal canal carcinoma, which occurs in less than 1% of cases, is often accompanied by severe complications. Surgery plays a crucial role in improving patients’ quality of life and controlling systemic paraneoplastic syndromes. This article reports a case of a 52-year-old female patient with recurrent anal canal squamous cell carcinoma (pT4bN0M0, Stage IIIB) 7 months after comprehensive treatment, complicated by rectovaginal fistula (RVF) and hypercalcemia of malignancy (HCM). Following multidisciplinary team (MDT) discussions, an innovative approach combining free omental packing with gracilis myocutaneous flap was adopted for the treatment of an isolated sacrococcygeal recurrent tumor after anal canal squamous cell carcinoma surgery. A tumor mass with a maximum diameter of 12.5 cm was completely resected. Postoperative pathological examination confirmed that no cancer cells were detected in multiple biopsies of the free resection margin, achieving R0 resection. After surgery, the patient's serum calcium level and serum squamous cell carcinoma antigen (SCC) returned to normal. Meanwhile, the bilateral combined myocutaneous flaps survived completely, RVF symptoms disappeared, the patient's quality of life was significantly improved, and the survival prognosis was enhanced.

## 1 Introduction

The incidence of anal carcinoma has shown an upward trend over the past decade, with squamous cell carcinoma being the most common histological subtype (1). Among patients with anal canal squamous cell carcinoma (SCC), 20%–30% present with refractory disease, and severe cases may experience recurrence within 2 years (2). Concurrent chemoradiotherapy is the main standard treatment for anal canal squamous cell carcinoma (3, 4). However, locally advanced recurrent lesions often invade adjacent organs (such as the vagina, urethra, and sacrum) and are complicated by rectovaginal fistula (RVF), which poses great challenges in clinical management and is associated with a poor prognosis (5). At this stage, extended resection may lead to extensive pelvic floor tissue defects, and the presence of infection, radiation-induced tissue damage, or postoperative scarring further complicates the repair and reconstruction process. Vascularized myocutaneous flap transplantation and free omental packing can provide well-vascularized tissue, effectively cover wounds, eliminate dead spaces, and promote wound healing, making them core techniques in surgical tissue reconstruction (6, 7). HCM is the most common Parathyroid syndrome, caused by bone metastasis or tumor secretion of parathyroid hormone-related protein (PTHrP). It is a sign of advanced disease and high tumor burden, and is significantly associated with shortened survival (8). This case report presents a rare patient with postoperative recurrence of squamous cell carcinoma of the anal canal, concurrent RVF and HCM. After MDT discussion, radical cytoreducsive surgery was performed, and the repair was carried out using a thin femoral myocutaneous flap and free omental tamping. The changes in blood calcium after the operation provided a basis for evaluating the success of the surgery.

## 2 Case data

**Figure 1:**
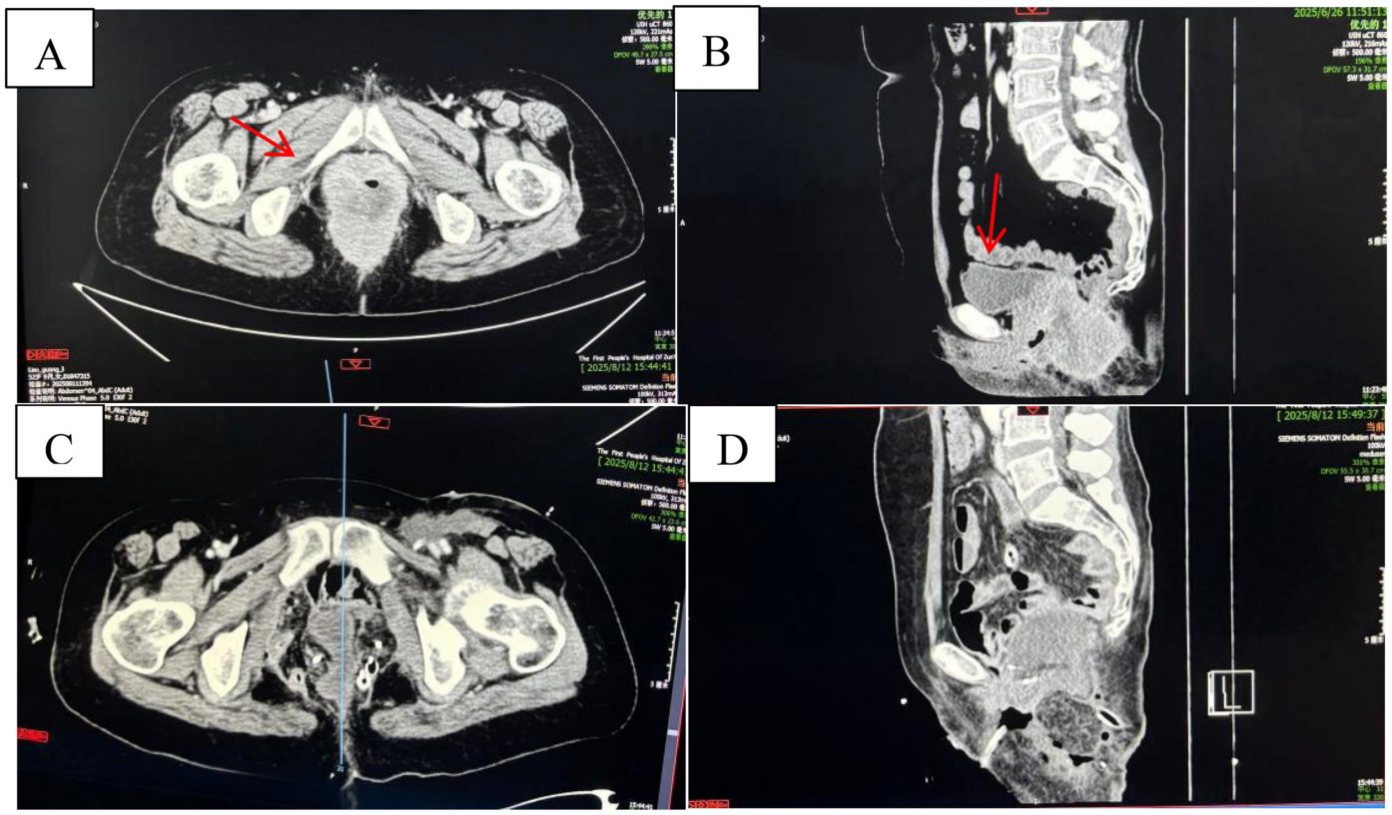
Pelvic CT: **(A)** preoperative (red arrow): An unevenly thickened soft tissue mass shadow was seen around the rectum-anal canal, with a maximum diameter of approximately 5.5 × 5.3 cm, unclear boundaries, uneven enhancement after contrast administration, and an unclear boundary with the adjacent vaginal wall. **(B)** Preoperative (red arrow): The bladder wall was smooth with uniform thickness, and no abnormal density lesions were found in the bladder cavity. **(C,D)** Postoperative: The irregular mass shadow around the rectum-anal canal was resected, and no other obvious abnormalities were observed.

**Figure 2:**
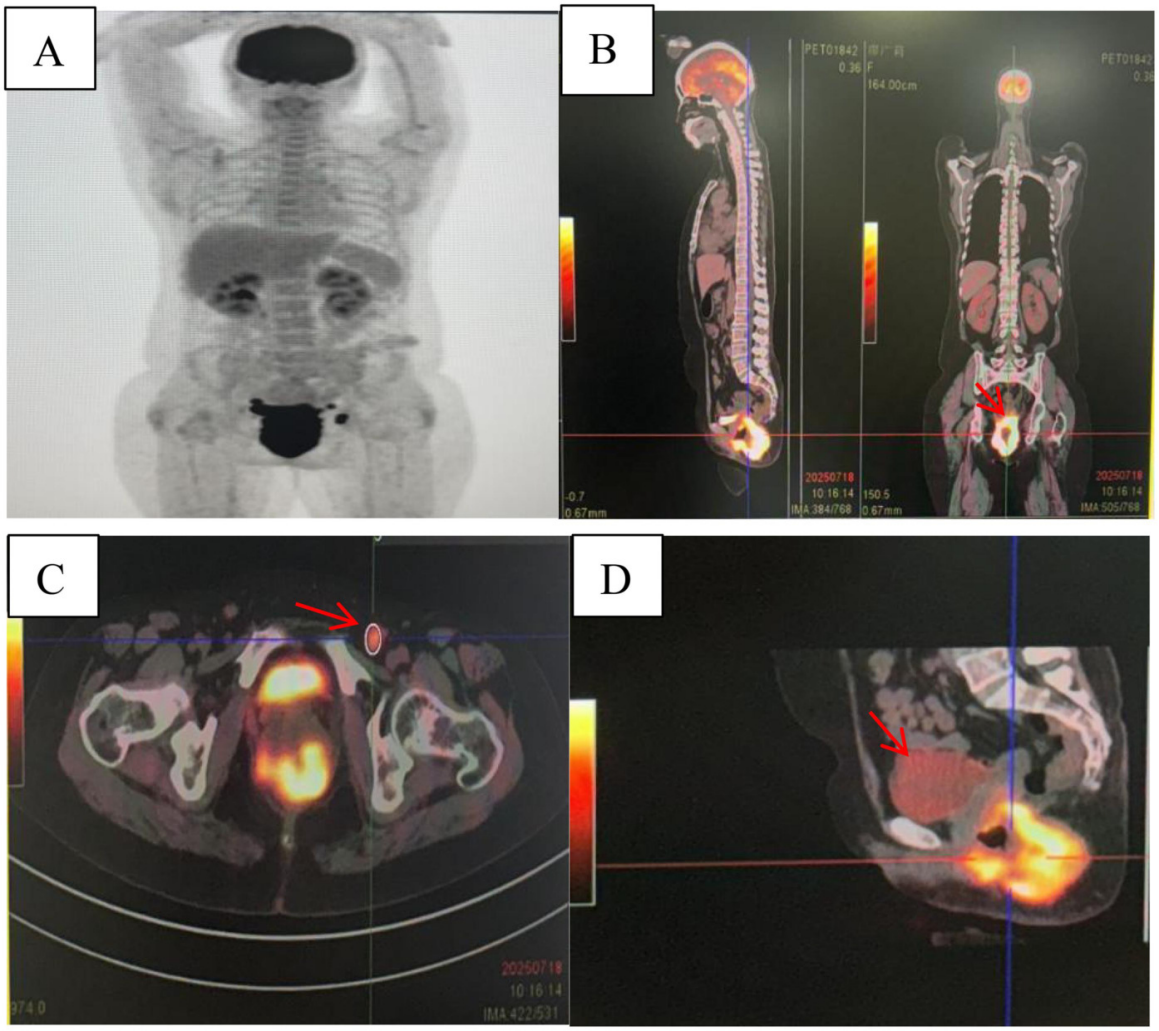
PET-CT: **(A)** fluorodeoxyglucose (FDG) accumulation was observed in the marked areas (black deposits). **(B)** Red arrow: Uneven thickening and soft tissue mass formation were seen around the rectum-anal canal, with an unclear boundary with the middle and lower segments of the vagina and abnormally increased FDG uptake. **(C)** Red arrow: Enlarged inguinal lymph nodes. **(D)** Red arrow: The bladder wall was not significantly thickened, and no abnormal density shadows were found inside; the distribution of F-FDG uptake was normal.

**Figure 3:**
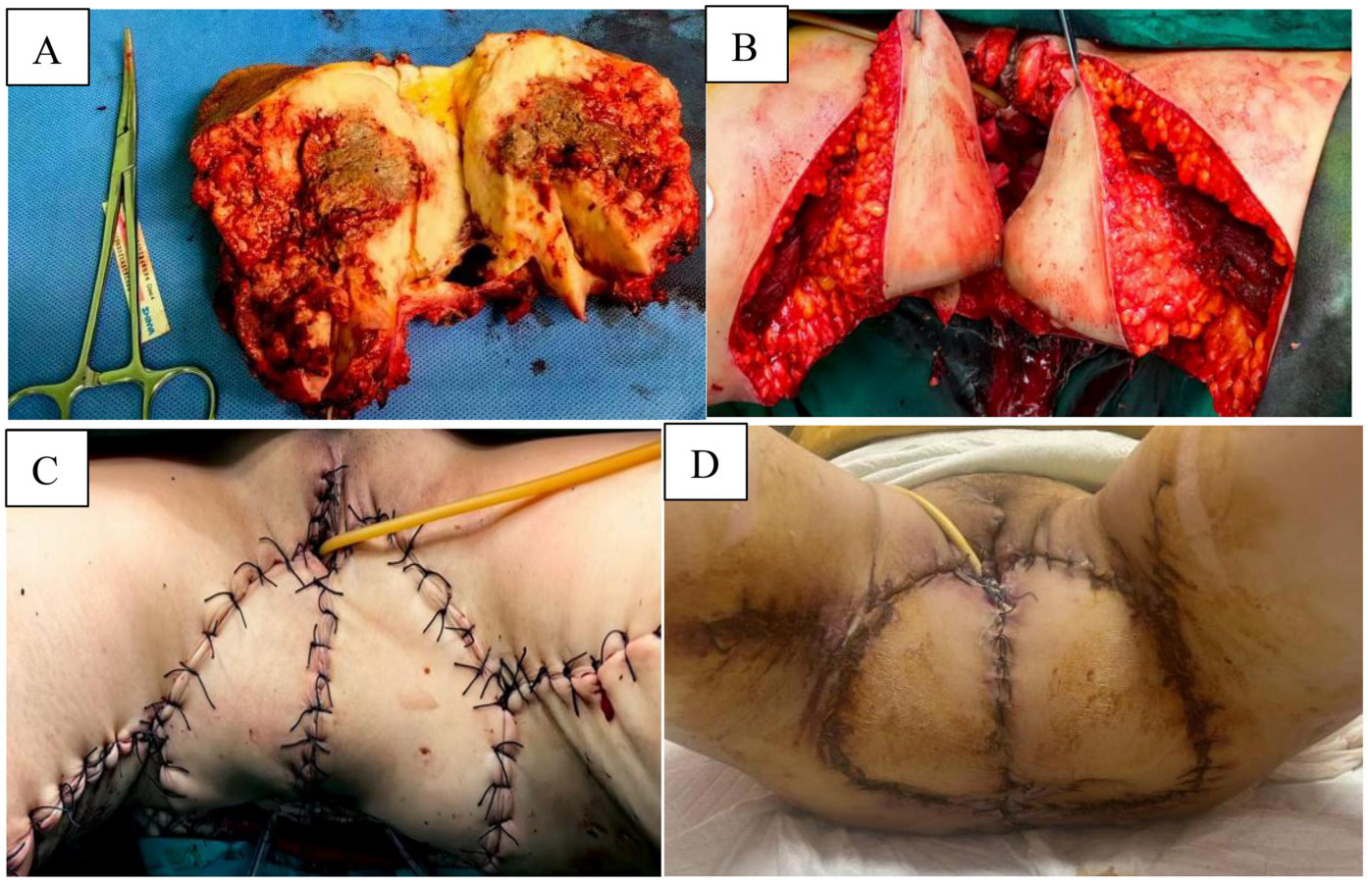
**(A)** Postoperative dissected specimen. **(B)** Intraoperative position and size of bilateral flaps. **(C)** Completed the suture of the surgical area after the operation. **(D)** Postoperative recovery of the patient during follow-up, 30 days after surgery.

This study obtained written informed consent from the patient and complied with the ethical requirements of the Declaration of Helsinki. A 52-year-old female patient was admitted to the hospital in July 2025 due to “recurrent anal distension for 1 year and abnormal vaginal discharge for 3 weeks”. Past Medical History: In October 2024, the patient was diagnosed with “rectal and anal canal tumor” at another hospital. Pelvic magnetic resonance imaging (MRI) revealed a space-occupying lesion in the lower segment of the rectum and anal canal, with invasion into the vagina. Consequently, the patient received one cycle of neoadjuvant chemotherapy with the CapeOX regimen. In November 2024, the patient underwent “laparoscopic radical resection of rectal cancer (Miles operation) + total hysterectomy with bilateral adnexectomy + resection and reconstruction of the posterior vaginal wall”. Postoperative pathological findings showed: (rectal) anal canal squamous cell carcinoma (moderately to poorly differentiated), measuring 5 × 4 × 3.5 cm, invading the full thickness of the anal canal and the posterior fornix of the vagina; no lymph node metastasis was detected (0/9); the tumor stage was determined as pT4bN0M0 [American Joint Committee on Cancer (AJCC) 8th Edition, Stage IIIB]. Starting from December 2024, the patient received 5 cycles of adjuvant chemotherapy with the CapeOX regimen combined with bevacizumab. Family History: There was no family history of hereditary diseases. Current Medical History: Three weeks before admission, the patient developed yellow, fecal-like vaginal discharge mixed with blood, accompanied by a foul odor, with an approximate volume of 10 mL per episode. Auxiliary Examinations-Enhanced pelvic CT (Figure 1) on admission: An irregular, contrast-enhancing soft tissue shadow was observed around the rectum-anal canal, which was larger than that in previous examinations and had an unclear boundary with the vaginal wall, suggesting tumor recurrence and possible RVF.PET/CT (Figure 2): Abnormally increased glucose metabolism was detected in the lesion, confirming tumor recurrence. The examination also showed vaginal invasion, RVF formation, and metastasis to pelvic and left inguinal lymph nodes. Physical Examination: A colostomy was visible in the left lower abdomen. Laboratory Examinations: Hypercalcemia: Serum calcium levels fluctuated between 2.59–2.94 mmol/L (Figure 3) (reference range: 2.11–2.52 mmol/L). Despite treatment with hydration, diuresis, and calcitonin, serum calcium levels remained elevated repeatedly. Serum squamous cell carcinoma antigen (SCC): >70.0 ng/mL. Routine bacterial culture of the rectovaginal fistula (RVF) discharge indicated positive results for Enterococcus faecalis and Acinetobacter baumannii. Consequently, cefoperazone was administered for anti-infection therapy. The patient's infectious symptoms were alleviated, which created favorable conditions for the subsequent surgery. Diagnosis: Postoperative recurrent anal canal squamous cell carcinoma, rectovaginal fistula (RVF), and tumor-related hypercalcemia.

**Figure 4:**
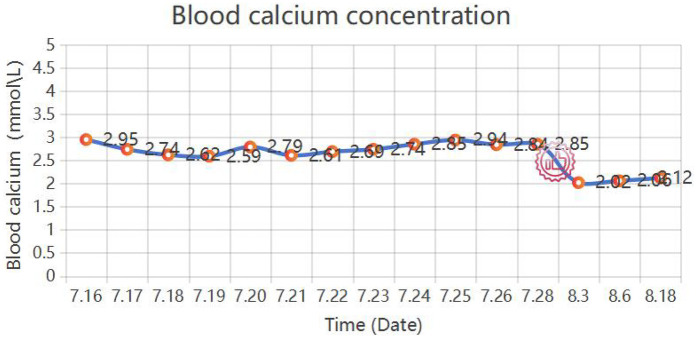
Changes in serum calcium concentration of the patient from admission to after surgery. The symbol indicates the operation time.

## 3 Treatment process

MDT discussion concluded that the patient had severe clinical symptoms and a large tumor burden, and HCM was directly caused by the tumor. Active surgical debulking and fistula repair were the only ways to control local symptoms and correct metabolic disorders. After symptomatic treatment and evaluation of the patient's general condition, in July 2025, the patient underwent “laparoscopic sacrococcygeal tumor resection + sacrum resection + total vaginal resection + extensive vulvectomy + inguinal and pelvic lymph node dissection + multiple biopsies of resection margins + free omental packing + bilateral pedicled gracilis myocutaneous flap transplantation”. However, based on preoperative and intraoperative evaluations (Figures 1, 2), complete resection of all sacrococcygeal tumors would result in an excessively large perineal defect. Preoperative pelvic MRI (Figure 2B) and PET-CT (Figure 2D) showed no tumor invasion of the bladder, and intraoperative exploration confirmed that the recurrent tumor had no adhesion to the bladder, only adhering to part of the sacrum. In addition, preoperative urodynamic examination indicated severe impairment of bladder detrusor function (maximum urine flow rate <5 mL/s, residual bladder volume >300 mL), and the bladder was assessed as having lost its urinary function. Considering that resection of the bladder and sacrum would lead to massive pelvic floor defects, which are prone to causing empty pelvis syndrome (9, 27) (such as pelvic organ prolapse, chronic pelvic pain, pelvic sepsis, fistula, and intestinal obstruction), the non-functional bladder was preserved intraoperatively to support the pelvic floor structure. Postoperatively, urine was drained through cystostomy, which ultimately reduced the incidence of postoperative complications such as pelvic organ displacement and infection.

For the massive anterior pelvic floor defect (with a postoperative defect area of approximately 18 cm × 25 cm) caused by tumor recurrence resection in this case, a single gracilis myocutaneous flap was considered to have risks of insufficient local tissue volume and incomplete dead space packing, while simple omental filling lacked the ability to cover the body surface wound. Therefore, based on the patient's condition, we innovatively adopted the surgical method of “bladder preservation + omentum + bilateral pedicled gracilis muscle combined repair to prevent postoperative empty pelvis syndrome”: First, the bladder was preserved to play a supporting role in the pelvic cavity; second, the omentum was freely filled into the deep dead space of the pelvic cavity, utilizing its advantages of rich blood supply, strong anti-infection ability, and strong secretory function to close the deep space and protect the posterior wall of the bladder; finally, bilateral 18 cm × 25 cm pedicled gracilis myocutaneous flaps and part of the adductor magnus muscle were used to cover the superficial wound. Compared with the single myocutaneous flap repair reported in the literature (10, 11), this surgical approach can improve the dead space filling rate. Additionally, the flap survival rate reached 100%, which significantly reduced the risk of complications such as postoperative infection and flap necrosis in complex pelvic floor defects. This provides a novel surgical option for the repair of similar massive anterior pelvic floor defects, especially in patients at high risk of infection.

Specific surgical procedures: During the operation, adhesions between the sacrococcygeal tumor and surrounding tissues were first dissected to clarify the tumor boundaries. The ultrasonic scalpel was used to gradually resect the involved sacrum, with care taken to avoid injury to the sacral nerves. When dissecting the tumor from the iliac blood vessels, vascular slings were applied to protect the internal and external iliac vessels, preventing massive intraoperative hemorrhage. After complete tumor resection, electrocoagulation was performed to achieve hemostasis at the sacral stump, which was then covered with hemostatic materials to reduce postoperative oozing. Flap transplantation: The patient was placed in the lithotomy position during the operation. Before harvesting the bilateral gracilis muscles, a pedicled flap consisting of the gracilis muscle and part of the adductor magnus muscle (18 cm in width and approximately 25 cm in length) was selected to ensure sufficient tissue volume (Figure 3B). The incision was made layer by layer, and the main vascular pedicles (such as the medial circumflex femoral artery) were identified. The gracilis muscle, part of the adductor magnus muscle, and the overlying skin were lifted together to form a large pedicled myocutaneous flap. The bilateral myocutaneous flaps were rotated to the massive perineal defect area: the muscle part was filled into the pelvic dead space, and the skin island part was sutured in alignment to reconstruct the vulvar shape and cover the perineal wound (Figure 3C). Two drainage tubes were placed in the pelvic cavity to create negative pressure, preventing fluid accumulation and blood stasis that could lead to infection. The donor site on the thigh was sutured directly.At the end of the operation, the patient was transferred to the Intensive Care Unit (ICU) for 2 days to allow close monitoring of vital signs and flap blood supply. For nutritional support, parenteral nutrition (glucose + amino acids + fat emulsion) was initially administered postoperatively, followed by a gradual transition to enteral nutrition (liquid diet). Regarding the anti-infection regimen, cefoperazone administration was continued after surgery, and discontinued once no infection was confirmed.

## 4 Results

### 4.1 Postoperative pathological examination

**Figure 5:**
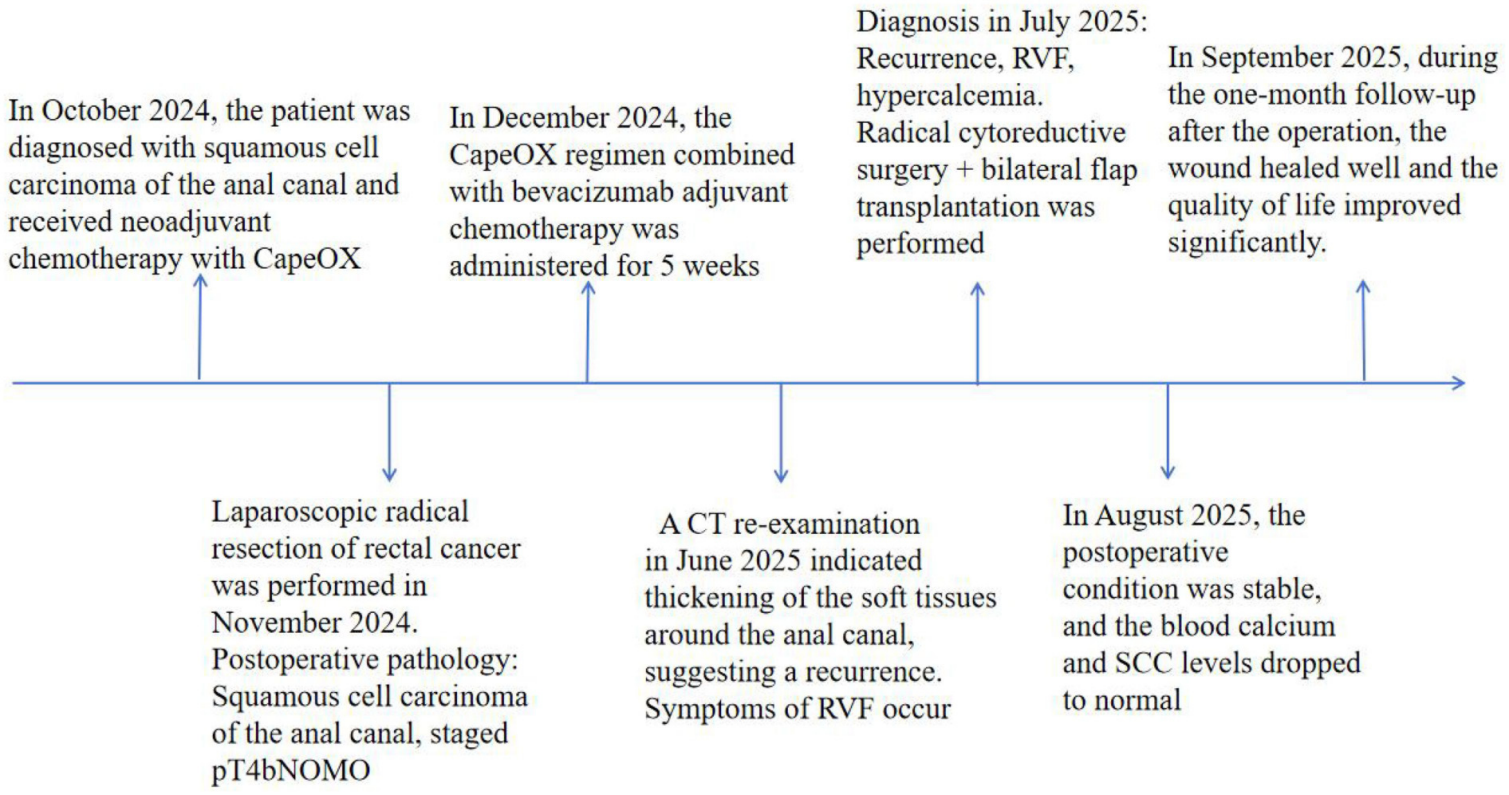
A timeline diagram of the patient's important treatments.

Dissected postoperative specimen (Figure 3A): The labial tissue of the sacrococcygeal tumor specimen was fragile, the pelvic floor skin tissue was hard in texture, and a cystic necrosis (measuring approximately 5 × 5 × 6 cm) was observed in the center of the dissected tumor. The surrounding tissue was hard and grayish-yellow.Postoperative pathology: 1. (Sacrococcygeal tumor) Squamous cell carcinoma (moderately differentiated), with a tumor size of approximately 12.5 cm × 10.5 cm × 9.5 cm, showing vascular and nerve invasion and involvement of the skin epidermis. Combined with the clinical history, it was considered to be involvement of (rectal and anal canal) squamous cell carcinoma (sacrococcygeal). Immunohistochemical results: CK pan (+), CK high molecular weight (+), CK5/6 (+), P40 (+), P63 (+), P16 (+), P53 (scattered +/wild type), EGFR (+), HPV (-), CD56 (-), CgA (-), Syn (-), Ki-67 (+, approximately 60%); 2. The submitted (tumor on the omentum) was a fibrous-wrapped fat necrosis nodule, with no tumor involvement; 3. No tumor involvement was found in the submitted (left wall and right wall) tissues; 4. Tumor involvement was detected in the submitted (right paraurethral) tissue, while no tumor involvement was found in the (left paraurethral, left posterior wall, and right posterior wall) tissues; 5. A small amount of tumor involvement was observed in the fibrous adipose tissue around the submitted (coccyx) tissue, with no tumor involvement in the bone tissue 6. Among the 7 submitted (inguinal) lymph nodes, 2 showed tumor metastasis (2/7). Postoperative Laboratory Examinations: The serum calcium level decreased rapidly after surgery. On the 3rd postoperative day, without calcium-lowering treatment, the serum calcium level dropped to 2.02 mmol/L spontaneously and remained within the normal range. The SCC level decreased to 1.9 ng/mL. Routine blood tests, C-reactive protein, and general bacterial culture results were all normal, and there was no infection or pelvic effusion after surgery. These changes in laboratory indicators strongly confirmed that the preoperative hypercalcemia was tumor-derived, and the surgery achieved effective tumor debulking. Postoperative Recovery: During the postoperative recovery period, the bilateral myocutaneous flaps had good blood supply and survived completely, and the RVF symptoms disappeared. After the patient's general condition improved, she was transferred to the oncology department for further treatment (such as radiotherapy and chemotherapy). Postoperative Follow-up: At the 1-month postoperative follow-up, re-examinations (including CT, serum calcium, and SCC) showed no abnormalities; the perineal wound healed well (Figure 3D), the flap texture was soft, and there was no redness, exudation, or necrosis. The patient's visual analog pain score decreased from 8 points preoperatively to 2 points, and the activities of daily living score increased from 45 points preoperatively to 85 points, indicating a significant improvement in quality of life. The patient was satisfied with the vulvar shape reconstruction effect, with no obvious scar contracture, normal lower limb movement function, and no pain or sensory abnormalities in the medial thigh. The patient's overall health status continued to improve, and follow-up will be continued for 1 year after surgery to observe long-term tumor control and complications. To fully reflect the important time nodes of the patient's treatment, a time node diagram was drawn (Figure 5).

## 5 Discussion

### 5.1 Treatment options for RVF

After Anal Canal Carcinoma Surgery RVF after anal canal carcinoma surgery is mostly caused by tumor recurrence and invasion or tissue necrosis after radiotherapy (12). It's obvious that clinical symptoms seriously affect the patient's quality of life and cause a huge psychological burden, but their treatment remains a clinical challenge. Currently, surgical treatment is the main approach (13); although there is no standardized surgical method worldwide, various specialized repair surgeries are available, mainly including pelvic packing (14), perineal rectal resection (15), biological mesh insertion (16), and tissue transposition surgery (17) (such as rectal mucosal advancement flap repair (18), Martius advancement flap and interventional plasty (19), and autologous fat injection (20). Most of these methods use flap transplantation, but due to poor local tissue blood supply (accompanied by infection and massive dead space), the failure rate of direct repair is extremely high (13). In addition, repair needs to achieve three goals: fistula closure, dead space packing, and wound coverage (6). Our department has previously performed radical surgery on patients with vaginal invasion after comprehensive treatment of rectal cancer and successfully repaired postoperative defects using gluteal muscle flaps (21). However, for the massive pelvic floor defect caused by tumor recurrence resection in this case, local flaps are difficult to meet the needs. Although free flaps can provide a large amount of tissue, they require a long operation time and carry high risks, making them unsuitable for patients with infection and poor general condition (7). Therefore, we considered adopting the strategy of “bladder preservation + free omental packing + bilateral pedicled gracilis muscle combined repair”: first, the bladder was preserved to play a supporting role in the pelvic cavity; then, the omentum was freely filled into the deep dead space of the pelvic cavity, and its advantages (rich blood supply, strong anti-infection ability, and strong secretory function) were used to close the deep space and protect the posterior wall of the bladder. The pedicled gracilis muscle (10, 22–24) was mainly used due to its rich blood supply and strong anti-infection ability: muscle tissue can effectively fill the massive dead space after tumor resection, and its rich blood supply provides a guarantee for resisting fecal contamination (an effect that cannot be achieved by simple flaps or skin grafting); in addition, the surgery can be completed in one stage, which saves time for subsequent treatment—since it is a pedicled flap, no microvascular anastomosis is required, and reconstruction can be completed in the same operation as tumor resection, shortening the total treatment time and reducing the patient's pain; finally, the damage to the donor site is minimal—the incision on the medial thigh can be sutured directly, with hidden scars and little impact on lower limb function. There are literature reports that the gracilis myocutaneous flap has a high success rate in vaginal reconstruction and pelvic floor reconstruction (11, 25), and its successful application in this case further confirms its reliability and effectiveness in addressing the most complex pelvic floor defects.

### 5.2 Tumor-related hypercalcemia

HCM is a common paraneoplastic syndrome (26), mostly caused by tumor secretion of parathyroid hormone-related protein (PTHrP) (27). It is a marker of advanced disease and massive tumor burden.Currently, multiple meta-analyses and systematic reviews have supported the association between tumors and serum calcium levels, confirming the correlation between tumor debulking surgery and reduced serum calcium (28–30). The patient's preoperative serum calcium level was as high as 2.94 mmol/L, which fully reflected the high activity and malignancy of the tumor (31). The serum calcium level returned to normal on the 3rd postoperative day. This significant change has important clinical significance: it serves as an extremely sensitive and specific “biological indicator” for evaluating the effectiveness of tumor debulking surgery (26). This change precedes any imaging evaluation, intuitively and strongly proving that this debulking surgery successfully removed the hormone-secreting source and achieved immediate success at the biochemical level, providing strong evidence for the decision-making value of this palliative surgery.

### 5.3 Importance of the comprehensive treatment model

It must be recognized that although surgery solved the urgent problems of RVF and hypercalcemia, this case already had lymph node metastasis, so the prognosis remains poor, and the patient still needs to be transferred to the oncology department for subsequent treatment after surgery. Surgery is part of the comprehensive treatment, aiming to remove obstacles for subsequent treatment and lay a good foundation. In the follow-up, the patient needs to rely on the MDT model and combine methods such as radiotherapy, chemotherapy, targeted therapy, or immunotherapy (3) to potentially prolong survival. This strategy—using surgery to solve current acute symptoms and create conditions for systemic treatment—is an important idea for managing such patients with advanced recurrence.

This study has the following limitations: ① It is a single-center, single-case report with a small sample size, and the generalizability of its conclusions needs to be further validated by multi-center, large-sample studies. ② Although the surgery achieved R0 resection (no cancer involvement at the resection margins) and lymph node metastasis was present, local symptoms and hypercalcemia were controlled in the short term; however, the long-term risk of tumor recurrence remains high. Currently, only 1 month of postoperative follow-up has been completed, and although short-term outcomes are favorable (flap survival, symptom relief, and normal serum calcium levels), we will strictly adhere to the follow-up plan to complete 1 year of postoperative follow-up, supplement examinations such as serum parathyroid hormone-related protein (PTHrP) detection and pelvic magnetic resonance imaging (MRI), and extend the follow-up period to 3–5 years. This will allow us to obtain long-term efficacy data and clarify the impact of surgery on patient survival. ③ The evidence for tumor-induced hypercalcemia is insufficient: Preoperative and postoperative serum PTHrP levels were not measured, and hypercalcemia was only inferred to be caused by tumor-secreted PTHrP based on indirect evidence, including changes in serum calcium levels, negative bone metastasis on positron emission tomography-computed tomography (PET-CT), and normal serum parathyroid hormone (PTH) levels. In subsequent cases, we will routinely measure serum PTHrP levels to clarify the association between tumors and PTHrP secretion. In the future, multi-center, large-sample, and long-term follow-up studies are expected to further optimize this treatment regimen and benefit more patients.

## 6 Conclusion

This case report confirms that under the guidance of the multidisciplinary team (MDT) model, radical debulking surgery combined with bilateral pedicled gracilis muscle and adductor magnus myocutaneous flap transplantation repair is a safe and effective diagnosis and treatment strategy for patients with locally advanced recurrent anal canal squamous cell carcinoma complicated by rectovaginal fistula (RVF) and tumor-related hypercalcemia (HCM). The surgery not only successfully resected the recurrent tumor (with a maximum diameter of 12.5 cm), achieved complete survival of the bilateral myocutaneous flaps, and eliminated RVF symptoms, but also enabled the preoperative intractable HCM to spontaneously return to the normal range on the 3rd postoperative day, with a significant decrease in serum squamous cell carcinoma antigen (SCC). It is proven that dynamic monitoring of serum calcium after surgery can be used as an immediate and objective biochemical indicator to evaluate the effect of tumor debulking.

Although the operation achieved R0, subsequent treatment is still required, this diagnosis and treatment plan still provides a key idea for solving local acute symptoms in such patients with advanced recurrence, improving quality of life, and creating conditions for systemic treatment. At the same time, it further verifies the reliability of the pedicled gracilis myocutaneous flap in repairing massive pelvic floor dead spaces and complex wounds, and provides an important reference for the clinical treatment of similar complex cases.

## Data Availability

The original contributions presented in the study are included in the article/Supplementary Material, further inquiries can be directed to the corresponding author.
